# Free-Field Hearing Test in Noise with Free Head Rotation for Evaluation of Monaural Hearing

**DOI:** 10.3390/jcm12227143

**Published:** 2023-11-17

**Authors:** Stanley Tetard, Caroline Guigou, Charles-Edouard Sonnet, Dhari Al Burshaid, Ambre Charlery-Adèle, Alexis Bozorg Grayeli

**Affiliations:** 1 Department of Otolaryngology-Head and Neck Surgery, Dijon University Hospital, 21000 Dijon, France; 2 ImViA, Laboratory of Imagery and Artificial Vision (EA 7535), Burgundy University, 21078 Dijon, France; 3 Amplifon Hearing Aid Center, 21000 Dijon, France

**Keywords:** binaural hearing, single-sided deafness, audiometry, head movements, stereophony, discrimination in noise, hearing rehabilitation

## Abstract

There is a discrepancy between the hearing test results in patients with single-sided deafness (SSD) and their reported outcome measures. This is probably due to the presence of two elements in everyday situations: noise and head movements. We developed a stereo-audiometric test in noise with free head movements to evaluate movements and auditory performance in monaural and binaural conditions in normal hearing volunteers with one occluded ear. Tests were performed in the binaural condition (BIN), with the left ear (LEO) or the right ear occluded (REO). The signal was emitted by one of the seven speakers, placed every 30° in a semicircle, and the noise (cocktail party) by all speakers. Subjects turned their head freely to obtain the most comfortable listening position, then repeated 10 sentences in this position. In monaural conditions, the sums of rotations (head rotations for an optimal hearing position in degrees, random signal azimuth, 1 to 15 signal ad lib signal presentations) were higher (LEO 255 ± 212°, REO 308 ± 208° versus BIN 74 ± 76, *p* < 0.001, ANOVA) than those in the BIN condition and the discrimination score (out of 10) was lower than that in the BIN condition (LEO 5 ± 1, REO 7 ± 1 versus BIN 8 ± 1, respectively *p* < 0.001 and *p* < 0.05 ANOVA). In the monaural condition, total rotation and discrimination in noise were negatively correlated with difficulty (Pearson r = −0.68, *p* < 0.01 and −0.51, *p* < 0.05, respectively). Subjects’ behaviors were different in optimizing their hearing in noise via head rotation. The evaluation of head movements seems to be a significant parameter in predicting the difficulty of monaural hearing in noisy environments.

## 1. Introduction

As the population ages and standards of living improve, deafness has become a real public health issue, with 1.5 billion people affected by hearing loss, according to the WHO in 2021 [1]. In the United States, the prevalence of single-sided deafness (SSD) is estimated at 0.11–0.14% [2]. Hearing impairment impairs general health, communication skills, social life and cognitive functions [3].

Binaural hearing is crucial in everyday life, providing improved hearing comfort for soft sounds, enhanced speech discrimination in noise, and the capacity to localize the sound source [4]. These binaural advantages are based on four mechanisms [4,5,6]: the binaural summation effect (a better detection of soft sounds), the squelch effect (extraction of signal from the background noise), suppression of the head shadow effect (omnidirectional hearing performances), and analysis of interaural time differences (ITD) and intensity (ITT), enabling azimuth localization, with an accuracy of 1 degree in the frontal sector and around 10 degrees in the lateral sectors [7].

These functions are impaired not only in single-sided deafness (SSD), but more extensively in asymmetric hearing losses (AHL, >20 dB of interaural difference in pure-tone average, PTA) [8]. Degraded spatial hearing with difficulties in sound source localization and speech perception in noise is at the basis of the auditory handicap in SSD (for review see [9]). In these SSD/AHL populations, the social and professional handicap is considerable, and the impact on perceived quality of life is all the greater as hearing loss increases [10]. In children, unilateral hearing loss deteriorates the development of speech and language, cognition, and more globally the quality of life (for a review, see [11]). The coping abilities of children are variable, and designing tests to detect those with difficulties would lead to early intervention and improved outcomes [11]. Hearing rehabilitation is therefore of major interest, with several strategies (the air and bone contralateral routing of signal (CROS) systems creating pseudo-stereophony, and cochlear implantation enabling binaural restitution approaching stereophony) [12].

As objective factors in rehabilitation success are difficult to individualize, it is worthwhile carrying out more specific assessments for SSD/AHL patients and estimating the prognosis for success as closely as possible [13]. Today, the most widespread principles for evaluating and monitoring rehabilitation, and the gains it brings, combine three criteria: free-field audiometry, speech audiometry in quiet and speech audiometry in noise. There are a multitude of tests (e.g., Hirsh, Matrix, and HINT) in which each variable (types and levels of signal and noise, number of speakers, and adaptive or fixed presentation) influences the results, and their choice must therefore guide the selection and interpretation of the test [14,15]. Speech in noise in a free-field condition appears to be the most ecological test since it is the closest condition to that of challenging everyday life situations. This category of tests generally provides us a signal-to-noise ratio at which a 50% discrimination is achieved (SNR50), together with specific normative values. However, this parameter is apparently insufficient to reflect the satisfaction of rehabilitated SSD subjects, because, despite the conclusive tests in the audiometry booth, 66% of patients do not adhere to the rehabilitation project [13], and there is great variability in subjective results, especially if the better ear is normal [16]. One limitation of all routine tests is that they are conducted with a fixed head position. There is a dynamic aspect of sound and noise azimuths in relation to the free head movements, which is overlooked in these tests.

Disposing of a method to evaluate the hearing handicap in SSD patients in conditions close to those of real life would potentially improve the selection of candidates for rehabilitation. Even if trials in real-life conditions can be organized for bone-anchored or CROS devices, such a test enhances the selection and reduces the cost. In the case of cochlear implantation, preoperative real-life trials are impossible, and predicting the functional outcome and patient satisfaction using an audiometric test is even more important. Moreover, the exploration of SSD in noisy and dynamic environments will probably reveal different coping strategies useful to adapt therapeutic education and hearing device designs or settings.

We hypothesized that the discrepancy between the routine audiometric tests and the patient’s reported outcome was probably due to the dynamic aspect of everyday life situations: noise fluctuating in intensity and azimuth, and head movements in search of the best position in relation to the signal source. Including these two elements in stereoaudiometry tests would enhance concordance with the patient’s outcome. We also hypothesized that SSD not only reduces speech discrimination in noise, but also increases head movements for sound source exploration.

The objective of this study was to develop a test combining free-field speech in noise audiometry and the recording of free head rotations in the search of the optimal position and to evaluate the test in normal-hearing subjects in binaural and monaural conditions.

## 2. Materials and Methods

### 2.1. Population

We conducted a prospective monocentric study in an otology tertiary referral center from June to July 2022, including 20 healthy adult subjects with normal hearing (no hearing difficulties and PTA < 20 dB on both ears). The participants were all French native speakers. Subjects with hearing symptoms, a neurological, rheumatological or orthopedic disorder preventing head and/or torso rotation movements, or a condition disrupting neurosensory abilities (including communication disorders) were excluded.

### 2.2. Study Materials

We used a soundproof audiometry booth, measuring 3 × 3 m, with 7 loudspeakers (Planet L, Elipson^®^, Champigny sur Marne, France) placed every 30° in a semicircle and numbered 1 to 7 from left to right. The subject was placed in the center, 1.0 m from the speakers, on a stool that could rotate freely but not translate. The speakers were located at the ear level.

The sound material (noise and voice) was broadcast using AmpliNext^®^ version 3.0 software (Amplifon^®^, Paris, France) [17], installed on a computer (ThinkPad^®^ Lenovo^®^, Intel^®^ (Beijing, China) core i5-4200U CPU 1.60 GHz, RAM 16.0 GB) fitted with an external sound card (Xonar^®^ U7 MKII, Asus^®^, Taipei, Taiwan), an audiometer (AC 40 Clinical Audiometer, Interacoustics^®^, Middelfart, Denmark) and an amplifier (STA-850D 8-channel, Monacor^®^, Bremen, Germany). QualiLoc^®^ v2.46 software (AMM ORL^®^, Massieux, France) was used to allocate the output for each speaker of noise alone or of a combination of voice and noise. For monaural conditions, a foam earplug (1100C30, 3M^®^, Maplewood, MN, USA) combined with a headphone (HDA300, Sennheiser^®^, Wedemark, Germany) occluded one ear.

HINT sentences (5 lists of 20 simple sentences) were administered as the speech material. Noise was defined by a cocktail party set at 60 dB (standardized ICRA noise, derived from the human voice, with the same frequency and time characteristics).

**Figure 1:**
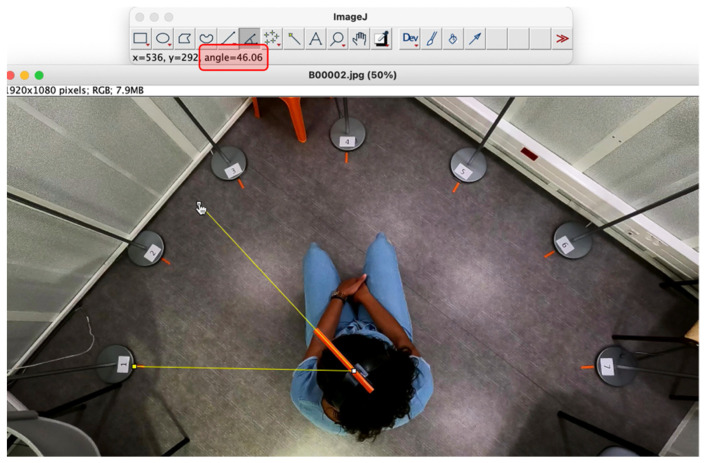
Experimental set-up and angle measurement using ImageJ^®^ version 1.41 software. Measurement of the angle between the voice-emitting speaker (speaker no. 1 in this example) and the position of the head. Orange rod: visual reference on the headband, perpendicular to the interaural axis. Red box: angle measured in degrees.

Video recordings were captured using a wide-angle camera (HERO9, GoPro^®^, San Mateo, CA, USA) mounted on the ceiling of the audiometry booth, vertically above the subject. A visual cue was placed on the subject’s head (headband fitted with a rod perpendicular to the interaural axis, Figure 1), to enable a computer analysis of the images.

Angles were measured via video analysis, manually using ImageJ^®^ software (freeware, version 1.41, available at https://imagej.nih.gov (accessed on 7 July 2022), NIH, Bethesda, MD, USA) [18]. By definition, a rotation of the head to the left in relation to the speaker of the voice defined a negative angle (0 to −180°) and a rotation to the right defined a positive angle (0 to 180°). Angles were measured at 5 s intervals, corresponding to the interval between each sentence presentation. All tests were carried out by the same examiner.

### 2.3. Study Process

After inclusion, the subject was examined via pure-tone audiometry. Subsequently, the SNR50 was determined in the binaural condition using the “Speech Perception In Noise (SPIN)” module (AmpliNext^®^), adaptively, in the free field with the signal delivered in front (speaker no. 4), and noise on all 7 speakers.

**Figure 2:**
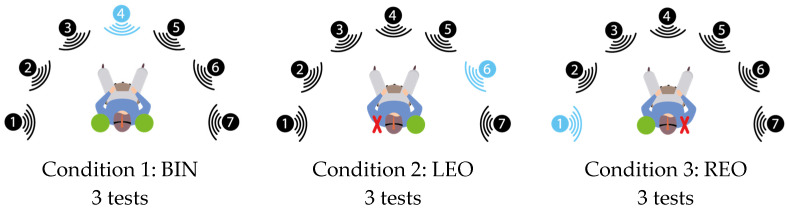
Test conditions. Condition 1: binaural (BIN); condition 2: left ear occluded (LEO); condition 3: right ear occluded (REO). Signal position (blue speaker) varied randomly for each of the 3 series in the 3 conditions. Noise was broadcasted by all 7 speakers (black and blue speakers).

Then, the participant went through 2 training series (10 sentences) in a free-field condition, noise condition, and free head rotation in binaural (BIN) condition. The training was followed by the test in the same conditions. Subsequently, the subject was trained with 2 series of 10 sentences in the same setting and with the left ear occluded (LEO), followed by the tests in LEO and right-ear-occluded (REO) conditions (Figure 2).

The subject then began the test in the free-field condition, noise condition and free head rotation condition, with 3 different measurement conditions: binaural (BIN), then left ear occluded (LEO) and finally right ear occluded (REO) (Figure 2).

In each condition, 3 tests were administered (S1–3, test–retests), with a different signal position for each. This position was defined randomly by the computer to avoid order effects. The SNR was fixed at the previously determined SNR50 level. Each subject performed 9 video-recorded series.

Each test was conducted as follows:

1. The subject started facing the central speaker (no. 4) and the noise was activated.
2. The signal was activated, and head position recording began;
3. The subject was then asked to rotate into the most comfortable position for optimal discrimination. In this search phase, as many sentences as necessary were presented, with a 5 s delay between each presentation;
4. The subject then maintained his head position until the end of the series and repeated the next 10 sentences;
5. At the end of the series, the subject was asked to give the number of the loudspeaker identified as the source of the signal.

For each series, the following parameters were recorded:

- During the exploration phase: the sum of rotations (in degrees), right and left boundary angles (relative to the signal azimuth, in degrees), amplitude of rotations (between right and left boundary angles, in degrees), final angle (relative to the signal azimuth, in degrees), and number of sentences required for the subject to find a comfortable listening position (number of presentations).
- After the exploration phase: the number of correctly repeated sentences (discrimination score out of 10) and the offset between the speaker identified by the subject as the source of the signal and the signal speaker.

After the tests, the subject evaluated the effortfulness of the test (0: no effortfulness; 10: very effortful) and its difficulty (0: easy; 10: very difficult), using a verbal rating scale.

### 2.4. Assessment Criteria

As the primary endpoint, we expected increased head movements during signal source exploration and lower discrimination scores in monaural conditions as compared to those in the binaural situation. Increased head movements were estimated via the sum of rotations during the exploration phase as described above.

The secondary endpoints were the correlation between the discrimination score and total rotation, the correlation between the difficulty and effortfulness VRS scores and total rotation, and the correlation between the discrimination score and total rotation.

### 2.5. Statistics

Power calculations were performed by G*Power (v. 1.3.6.9, Heinrich Heine Universität Düsseldorf, Düsseldorf, Germany) [19]. Based on stereoaudiometry studies in patients with rehabilitated unilateral hearing loss [20], a reduction in noise discrimination score of 20% with an occluded ear compared to that in the binaural condition and an inter-individual performance variation of 10% were anticipated. Defining β = 0.05 and α = 0.05, for an ANOVA comparison with repeated measures between factors with 3 subgroups (BIN, LEO and REO), 15 subjects were required. We set the number of participants at 20 to compensate for subjects who might not have completed all the tests.

Statistical tests were carried out using Prism software (version 8, GraphPad Software^®^, San Diego, CA, USA). Quantitative variables were described by their mean ± standard deviation [minimum; maximum].

Parameters related to head position and auditory performance did not follow a normal distribution (Kolmogorov–Smirnov test, *p* < 0.01 to *p* < 0.001). Comparisons of these different positional parameters (left angle, right angle, amplitude, and total rotation) or auditory performance parameters (discrimination score; localization score) between the different conditions (BIN, LEO and REO) were performed using an ANOVA test or a mixed model with a correction for the absence of sphericity according to Geisser–Greenhouse and a correction for multiple comparisons according to Tukey.

Comparisons between test and retests (S1 to S3) for the same parameter were carried out using a non-parametric Friedmann repeated-measures test, followed by Dunn multiple-comparison correction.

Significance levels were defined as follows: *p* > 0.05: non-significant; *p* < 0.05: significant; *p* < 0.01: highly significant; *p* < 0.001: very highly significant.

The correlation between different series of the same condition for head position or auditory performance parameters was estimated using Pearson’s r: r < 0.40: poor correlation; 0.40 < r < 0.59: moderate correlation; 0.6 < r < 0.74: good correlation; 0.75 < r < 1: excellent correlation [21].

## 3. Results

### 3.1. Population

All 20 subjects completed the tests. The sex ratio was 1, with an average age of 28.4 ± 5.1 years [22.4; 44.8]. As evaluated via pure-tone audiometry, the mean hearing threshold was 9.9 ± 2.9 dB [5.0; 14.3] on the right, and 10.5 ± 3.6 dB [5.0; 17.1] on the left. The mean interaural difference was 2.8 ± 2.1 dB [0.0; 7.1]. The mean SNR50 was 58.5 ± 2.3 dB [52; 62], and the mean SNR was −1.6 ± 2.3 dB [−8.0; +2] with the normalized cocktail party noise set at 60 dB.

### 3.2. Head Position

The parameters describing the head position showed significant dispersion, even in the binaural condition (Table 1). This suggested that, even in individuals with normal hearing, strategies for exploring sound space in binaural and monaural conditions vary significantly from one individual to another.

**Figure 3:**
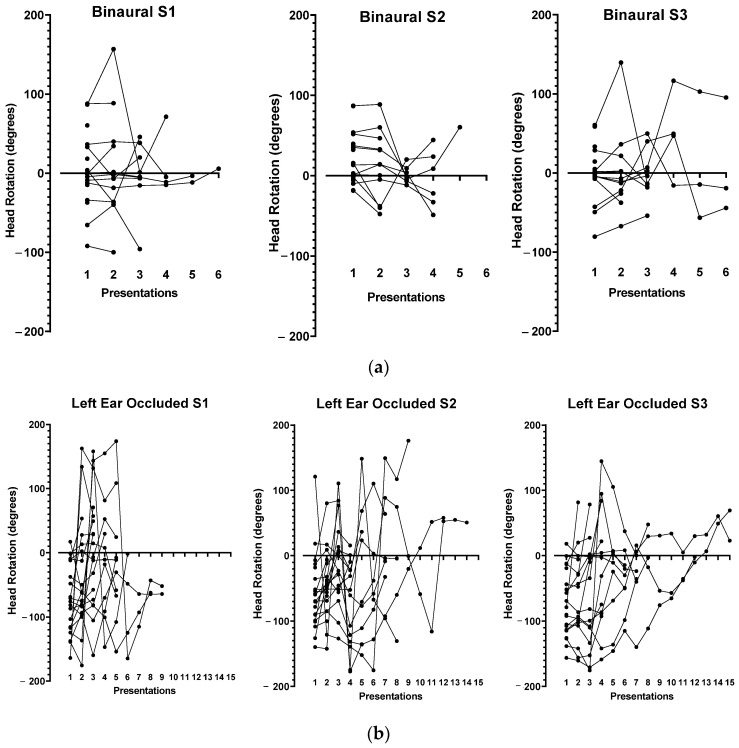

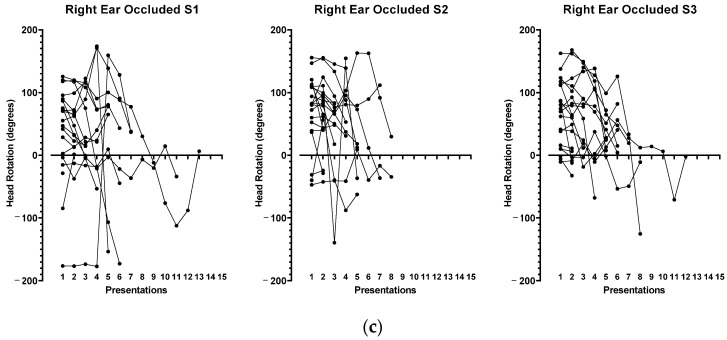
(**a**). Head position as a function of presentations during the exploration phase for the 3 tests (S1–S3) in the binaural condition. (**b**). Head position in relation to the number of presentations during the hearing comfort position search phase for the 3 series of measurements (S1–S3) in the LEO condition. (**c**). Head position in relation to the number of presentations during the hearing comfort position search phase for the 3 series of measurements (S1–S3) in the REO condition.

**Figure 4:**
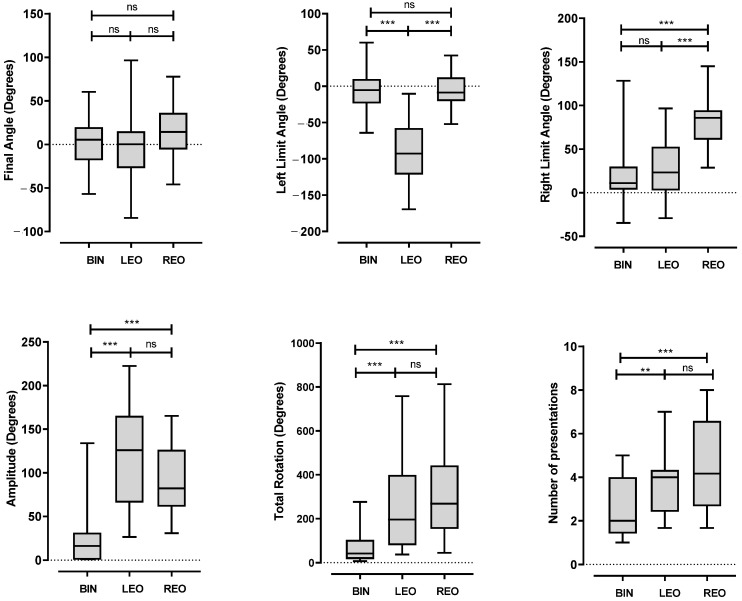
Head position parameters during and at the end of the search phase for the 3 test conditions (BIN, LEO et REO). Box-and-whisker plots show the median (horizontal line), 1st and 3rd quartiles (gray box) and the minimum and maximum (whiskers). Values in each condition are the mean of the 3 test replicates in 20 subjects. Angles are measured relative to the signal azimuth. ns: not significant; ** *p* < 0.01 and *** *p* < 0.001; 2-way repeated-measures ANOVA test followed by Tukey correction for multiple comparisons.

As expected, during the exploration phase, total rotations were greater with one occluded ear than those in the binaural condition (LEO 254.7 ± 212.0 and REO 308.4 ± 208.0 versus BIN 74.30 ± 75.50, *p* < 0.001, ANOVA, Figure 3a–c and Figure 4). There was no difference in the sum of rotations or amplitude between the REO and LEO conditions (ANOVA, not significant, Figure 4).

A comparison of test–retests (S1–S3) in the same condition shows no learning effect. Indeed, there was no decrease in the total rotation or amplitude with the repetition of the series (Table 1). However, there was a good interclass correlation between test and retests for amplitude and total rotation in the BIN condition. In monaural conditions (LEO and REO), the correlation between series disappeared for total rotation and amplitude, suggesting a lack of strategy for head positioning in our experimental conditions.

It should be noted that, in the LEO condition, the left limit angle is lower than the one measured in the REO and BIN conditions, and, symmetrically, in the REO condition, the right limit angle is higher than that in the LEO and BIN conditions (Figure 4). This suggests a more extensive exploration of the auditory field by rotating the head on the side of the occluded ear, with the aim of optimizing sound detection by the only available ear. The values of these angles show good inter-class correlations (left angle in the LEO condition and right angle in the REO condition), suggesting the implementation of a strategy by the subjects (Table 1).

Meanwhile, during the exploration phase, the number of presentations was higher in the LEO (3.9 ± 1.51 [1.67; 7]) or REO (4.38 ± 2.05 [1.67; 8]) conditions in comparison to that in the BIN condition (2.50 ± 1.33 [1; 5]), while there was no difference between the LEO and REO conditions (Figure 4).

### 3.3. Hearing Performances

The average discrimination score in the BIN condition was 7.7 ± 1.45 [5; 10]. As expected, this score was lower in LEO (5.1 ± 1.18 [3; 7], *p* < 0.001 versus BIN) and REO (6.8 ± 1.39 [4.7; 9.3], *p* < 0.05 versus BIN) conditions. There was no difference in scores between LEO and REO conditions (not significant, repeated-measures ANOVA followed by Tukey). We found a moderate test–retest correlation in BIN, but no correlation in LEO or in REO conditions (Table 2).

There was no correlation between the discrimination score and total rotation in binaural (Y = 15.13 × X − 42.23, R2 = 0.08, Pearson r = 0.29, not significant) and monaural conditions (Y = 65.64 × X − 56.54; Y: total rotation in degrees; X: discrimination score R2 = 0.10, Pearson r = 0.31, not significant), suggesting the independence of search movements and auditory performance.

### 3.4. Localization

In the BIN condition and in all three test replicates, the speaker delivering the voice material was correctly identified 58 times (97%, *n* = 60), with an offset of one speaker in only two occasions (mean offset: 0.03 ± 0.18 speaker). In contrast, in the LEO condition, the signal source was correctly identified in only 26 cases (43%, *n* = 60), with an error of one to three speakers in the remaining 34 cases (mean offset: 0.65 ± 0.66 speaker). Similarly, in the REO condition, the signal source was correctly identified 31 times (52%, *n* = 60), with an error of one to three speakers in the remaining 29 cases, (mean offset: 0.63 ± 0.78 speaker). Mean localization errors in the LEO and REO groups were higher than those in the BIN condition (*p* < 0.001, Friedman’s test, followed by a correction for multiple comparison according to Dunn). Offsets were not significantly different between the LEO and REO conditions, (*p* = 0.98, Freidman’s test).

The direction of the shift between the presumed speaker and the actual source of the voice was indifferent and did not differ between the LEO and REO situations (*p* = 0.65, Friedmann’s test).

In the BIN condition, there was a moderate correlation between localization errors between series. In monaural conditions, the test–retest correlations were insignificant (except for LEO, S1–S3), indicating the randomness of source detection (Table 3).

In the monaural condition, localization errors were not correlated with discrimination scores (Pearson r = −0.12, not significant) or total rotation (Pearson r = 0.38, not significant).

### 3.5. Subjective Evaluation of the Test

Subjects rated the difficulty of the test at 5.20 ± 1.54 [2; 8] and the effortfulness at 3.40 ± 2.28 [0; 8] out of 10. There was a good correlation between these two scores (Pearson r = 0.63, *p* < 0.01).

**Figure 5:**
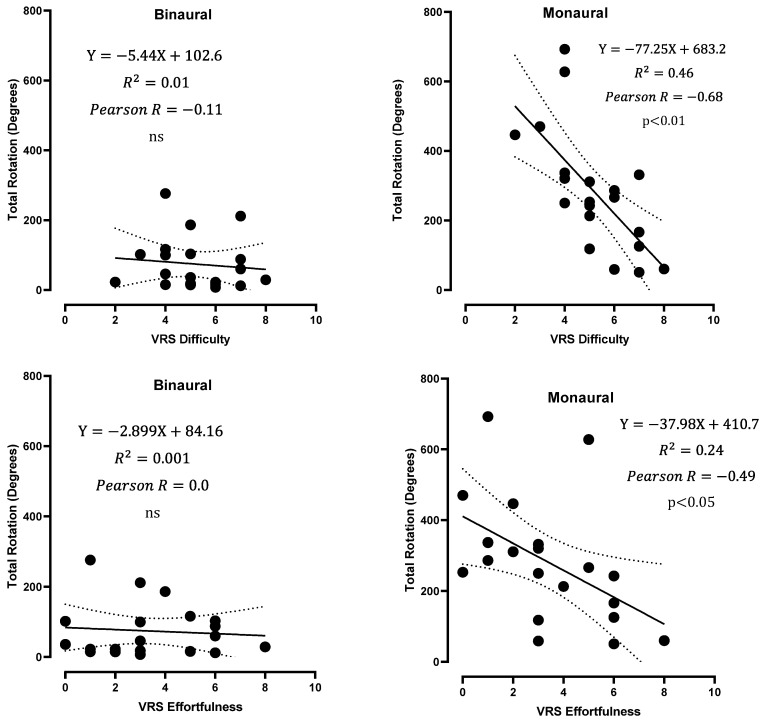
Total rotation as a function of the effortfulness and difficulty VRS scores in binaural and monaural conditions (average of LEO and REO values). Linear regressions with 95% confidence interval bands (dashed) are shown, along with Pearson’s r and the *p*-value; ns: not significant.

**Figure 6:**
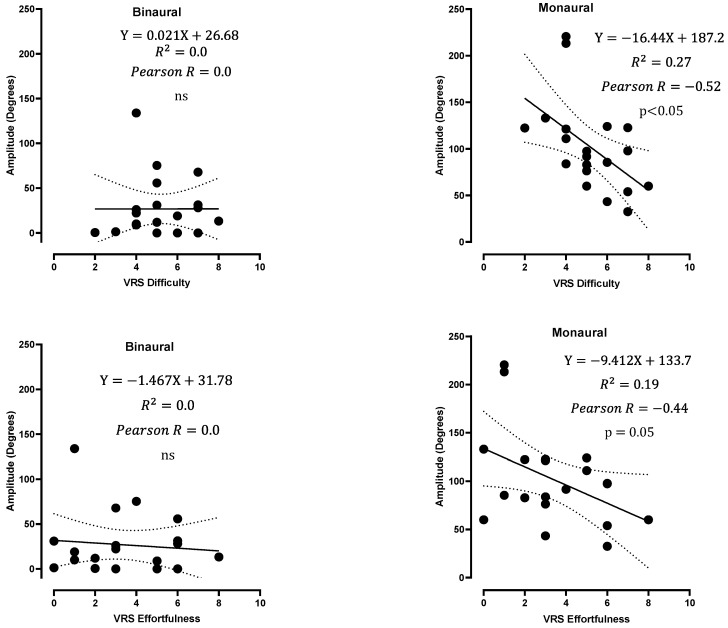
Rotation amplitude as a function of effortfulness and difficulty VRS scores in binaural and monaural conditions (average of LEO and REO values). Linear regressions with 95% confidence interval bands (dotted lines) are shown, along with Pearson’s r, ns: not significant.

Difficulty and effortfulness scores were negatively correlated with the total rotation and amplitude of rotation in the monaural condition (mean of LEO and REO values) but not in the BIN condition (Figure 5 and Figure 6), suggesting that patients with greater exploratory movements were less bothered with an occluded ear and found the test easier.

**Figure 7:**
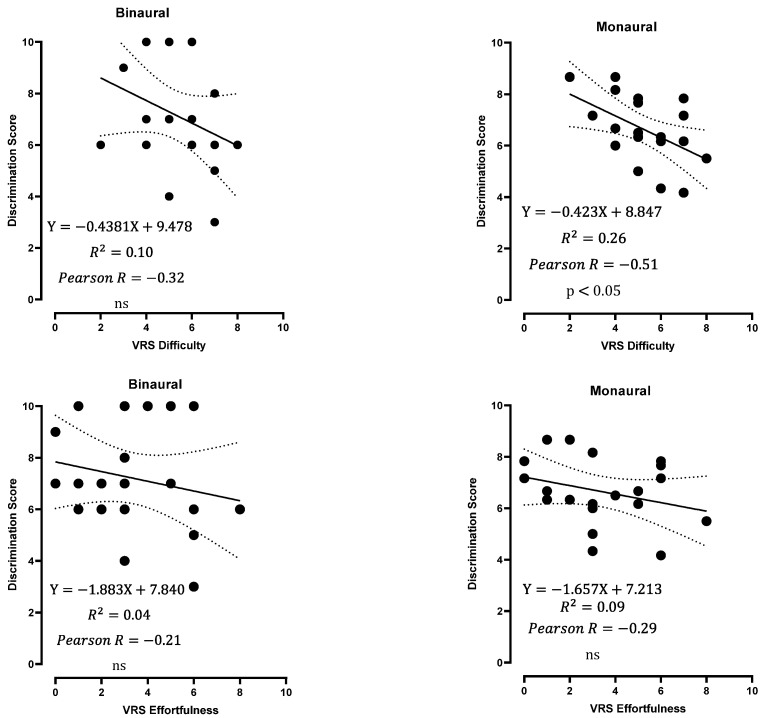
Discrimination score in relation to the VRS of effortfulness and difficulty in binaural (BIN) and monaural conditions (average of LEO and REO values). Linear regressions with 95% confidence interval bands (dotted lines) are shown, along with Pearson’s r. ns: not significant.

Furthermore, the difficulty score was negatively correlated with the discrimination score in the monaural condition (Figure 7), while the effortfulness score was not. These results suggest that head movements are as important as the discrimination score in assessing auditory difficulty. Difficulty and effortfulness scores were not correlated with localization errors in the monaural condition (Pearson r = −0.32 and −0.37, respectively; not significant).

## 4. Discussion

Given the low relative prevalence of unilateral deafness [2], and the intricacy of the factors involved in hearing impairment [13,22], it is difficult to identify factors influencing daily discomfort and patients’ interest in rehabilitation. However, this assessment is essential to the rehabilitation process.

In this study, we showed that a stereoaudiometric test performed in noise with free head movements can discriminate between monaural and binaural situations. No ceiling or floor effect was observed for any of the parameters studied. We showed that in monaural situations, head movements in search of the optimal listening position were greater, and the discrimination score was lower, than those in binaural situations. These two parameters appeared to be independent, but were both correlated with test difficulty, suggesting that a combined score adding parameters related to head movements and those pertaining to speech discrimination in noise might better account for the day-to-day difficulties of patients with unilateral deafness.

When measuring binaural prosthetic gain, the improvement in SNR cannot remain the only objective element in monitoring the hearing in noise conditions of rehabilitated patients [12]. Indeed, despite an objective improvement of hearing in noise conditions in existing tests, many patients abandon their hearing aids after a few months’ use [13]. This can be explained by the fact that, in everyday life, we do not choose the loudness of the situation in which we find ourselves, that signal and noise sources vary rapidly in time and space, and that probably, some subjects cannot adapt quickly to these variations, failing to position themselves optimally in relation to the sound source. In our study, by setting a SNR at the SNR50 level of each subject, we aimed to homogenize auditory difficulty, observing only rotational movement as the only variable in the subject’s adaptation to its environment, as well as any strategies implemented during the test.

The study of head movements during a free-field audiometric test with several sound sources is an original and innovative approach, as most existing audiometric tests are performed with the head fixed, and with varying loudspeaker configurations and volume [14]. These tests evaluate the contribution of the hearing aid in the most unfavorable situation (noise on the side of the functional ear and signal on the deaf side), and often serve to demonstrate the system to the patient. However, turning the head physiologically improves the distinction of signals of interest, through binaural unmasking [4]. In healthy subjects, proper head orientation can improve the SNR for understanding speech in noise conditions by up to 8 dB [23]. In bilateral cochlear implant patients, front/back confusion in sound source localization is markedly reduced when head movements are allowed, suggesting that the true hearing acuity of this population is underestimated by conventional static tests [24].

An important aspect of testing monaural hearing or hearing in noise is the effect of training. Several authors have shown that the capacity of pure-tone azimuth localization improves with training [25,26,27]. A higher dependency on spectral cues and sensitivity central adjustment in the case of asymmetrical hearing [25] and enhanced spatial performances with the exploitation of visual cues [26] have been advocated. Hearing performances in noise also improve with different types of training such as hearing in noise [27] conditions or even musical activities [28,29]. These issues represent a potential limit for the interpretation of the results in participants with no previous experience of hearing loss even if they have been trained before the trials and even if test–retests did not show a training effect on any measured parameter.

During the exploration phase, when subjects sought the optimal head position, we could imagine that subjects with smaller rotations would express less difficulty and effortfulness, because they had a greater ability to find the optimal position quickly. On the contrary, we were surprised to find that subjects with greater head movements found the test easier and less arduous. This reduction in the ability to find a comfortable hearing position in a monaural situation could explain the reported discomfort and the motivation for hearing aid fitting in the case of unilateral deafness, yet this ability is not explored in current tests [30].

In a binaural situation, even in the presence of noise, subjects had excellent signal source localization performances and a near-zero error rate, whereas in a monaural position, errors were frequent, as predicted in multiple previous studies [31,32,33]. However, these error rates were neither correlated with the discrimination score, nor with the difficulty and effortfulness scores. Although it is important to detect or pay attention to the signal azimuth to position oneself optimally (typically between 0 and 30 degrees for the binaural subject, for maximum binaural summation and unmasking) [34], this lack of correlation suggests that in overall difficulty, the ability to position oneself optimally relative to the signal source prevails over the ability to locate it accurately. Moreover, in patients with unilateral profound deafness, localization is rarely improved by pseudo-binaural rehabilitation and therefore does not appear to be a relevant criterion for judging the impact of hearing rehabilitation.

In order to measure only the auditory performance of subjects and improve the sensitivity of measurements, some authors suggest carrying out tests in the dark or blindfolded [35,36]. This hampers comprehension and communication as a whole, with a loss of visual information necessary for normal exchanges in everyday life (body gestures, lip-reading, facial mimics, etc.) [33]. As the aim of our test is to approximate real-life conditions, this configuration was not adopted. The results would probably have been underestimated by removing additional cues from the listener, such as the position of the loudspeakers. This must contribute to the fact that, in our study, the final position of the subjects relative to the source showed no significant difference between groups, although logically, we would have expected a spontaneous orientation of the non-occluded ear towards the source loudspeaker to increase the subject’s discrimination.

Among the objective factors predictive of rehabilitation success, age is often mentioned [37]. As the population studied in our test was relatively young (mean age: 28.4) and homogeneous, it would be interesting to replicate this test on a wider range of age, to explore a possible correlation between head movements and the age of the subjects.

For the same level of deafness, the extent of hearing impairment depends not only on audiological factors, but also on non-auditory criteria (culture, lifestyle, associated impairments, etc.) [38]. Thus, the evaluation of rehabilitation is also based on subjective criteria, and the test developed in our study does not preclude the use of quality-of-life questionnaires, whose specificity and sensitivity have proved their validity, either in general, such as APHAB [39] or SSQ [40], or more specifically developed for monaural situations such as BBSSD [22].

In conclusion, the strategy of head movements to optimize hearing in noisy environment varies between subjects and is probably a significant factor in patient-reported outcomes in SSD. The combination of the two parameters identified in our study, discrimination score and head rotation, in a composite score, would be an interesting avenue through which to predict the outcome of rehabilitation in SSD patients.

## Figures and Tables

**Table 1:** Head position parameters in degrees for the 3 test conditions and 3 replicates (S1–S3).

		S1	S2	S3	Pearson R, *p*-Value
BINAURAL	Final angle	1.8 ± 47.47 [−99.9; 88.50]	9.1 ± 37.70 [−49.0; 86.7]	1.5 ± 35.78 [−54.0; 95.4]	S1–S2: 0.383 ns S1–S3: 0.37 ns S2–S3: 0.23 ns
	Left limit	−10.7 ± 42.00 [−99.9; 87.9]	0.9 ± 35.66 [−49.0; 86.7]	−10.9 ± 30.50 [−80.4; 60.7]	S1–S2: 0.5 ** S1–S3: 0.43 ns S2–S3: 0.05 ns
	Right limit	15.9 ± 50.99 [−91.9; 156.8]	23.7 ± 31.63 [−18.5; 88.8]	20.0 ±45.69 [−54.0; 139.6]	S1–S2: 0.002 ns S1–S3: 0.05 ns S2–S3: 0.20 ns
	Amplitude	26.6 ± 38.41 [0; 155.7]	22.8 ± 28.71 [0; 93.1]	30.9 ± 46.10 [0; 153.2]	S1–S2: 0.76 *** S1–S3: 0.68 *** S2–S3: 0.67 **
	Total rotation	76.3 ± 85.30 [1.0; 316.0]	65.6 ± 59.04 [3.7; 254.2]	81.0 ± 109.9 [1.0; 388.1]	S1–S2: 0.78 *** S1–S3: 0.58 ** S2–S3: 0.75 ***
LEFT EAR OCCLUDED	Final angle	−3.2 ± 55.89 [−72.9; 108.5]	0.2 ± 71.68 [−135.2; 176.1]	−5.0 ± 61.15 [−125.7; 84.0]	S1–S2: 0.22 ns S1–S3: −0.16 ns S2–S3: 0.38 ns
	Left limit	−97.0 ± 54.25 [−175.5; −11.6]	−85.6 ± 52.71 [−177.0; −6.7]	−87.9 ± 53.45 [−175.4; −5.4]	S1–S2: 0.48 * S1–S3: 0.80 *** S2–S3: 0.29 ns
	Right limit	36.4 ± 73.6 [−72.9; 174.0]	35.1 ± 64.91 [−99.9; 176.1]	11.57 ± 71.58 [−125.7; 144.7]	S1–S2: −0.21 ns S1–S3: −0.29 ns S2–S3: 0.06 ns
	Amplitude	133.4 ± 108.40 [13.9; 338.6]	120.6 ± 99.52 [0; 351.4]	99.4 ± 83.40 [0; 320.2]	S1–S2: 0.05 ns S1–S3: 0.135 ns S2–S3: 0.06 ns
	Total rotation	341.5 ± 297.30 [46.0; 1107.0]	380.4 ± 373.02 [17.9; 1277.0]	355.3 ± 351.90 [5.9; 1468.0]	S1–S2: 0.28 ns S1–S3: 0.73 *** S2–S3: 0.26 ns
RIGHT EAR OCCLUDED	Final angle	2.2 ± 69.29 [−172.8; 78.1]	30.0 ± 56.6 [−62.6; 154.5]	7.7 ± 47.9 [−125.3; 90.5]	S1–S2: −0.18 ns S1–S3: 0.30 ns S2–S3: −0.24 ns
	Left limit	−26.0 ± 78.00 [−177.2; 71.0]	5.6 ± 60.01 [−139.4; 83.4]	−5.7 ± 46.7 [−125.3; 64.1]	S1–S2: 0.23 ns S1–S3: 0.21 ns S2–S3: −0.09 ns
	Right limit	76.9 ± 58.22 [−28.7; 173.9]	88.5 ± 51.03 [−24.4; 162.9]	78.1 ± 52.92 [−5.8; 167.8]	S1–S2: 0.57 ** S1–S3: 0.56 ** S2–S3: 0.49 *
	Amplitude	102.9 ± 107.90 [0; 336.5]	82.9 ± 79.19 [1.0; 294.0]	83.8 ± 67.02 [0; 265.1]	S1–S2: 0.30 ns S1–S3: 0.27 ns S2–S3: 0.12. ns
	Total rotation	330.1 ± 319.80 [28.7; 1034]	318.6 ± 212.0 [55.4; 781.4]	288.0 ± 249.1 [1.5; 876.4]	S1–S2: 0.29 ns S1–S3: 0.12 ns S2–S3: 0.01 ns

Values are expressed in mean ± standard deviation [min; max], *n* = 20. Correlation is assessed via Pearson’s r and *p*-value. ns: not significant; * *p* < 0.05, ** *p* < 0.01, and *** *p* < 0.001.

**Table 2:** Discrimination scores for the 3 test conditions (EF, LEO and REO) and the 3 measurement series (S1–S3).

Condition	S1	S2	S3	Correlation
**BIN**	7.2 ± 2.09 [3; 10]	8.35 ± 1.73 [5; 10]	7.6 ± 1.85 [5; 10]	S1–S2: 0.17 ns S1–S3: 0.46 * S2–S3: 0.53 *
**LEO**	6.4 ± 2.23 [1; 10]	6.8 ± 2.15 [3; 10]	6.3 ± 2.73 [0; 10]	S1–S2: 0.07 ns S1–S3: 0.47 * S2–S3: 0.24 ns
**REO**	7.1 ± 2.06 [4; 10]	7.1 ± 1.83 [4; 10]	6.4 ± 2.18 [2; 10]	S1–S2: 0.11 ns S1–S3: 0.15 ns S2–S3: 0.39 ns

Values are expressed in degrees mean ± standard deviation [min; max], *n* = 20. Correlation is assessed via Pearson’s r and *p*-value. ns: not significant, * *p* < 0.05. There is no difference between S1, S2 and S3 for any parameter in the 3 conditions (Friedmann’s non-parametric repeated-measures test).

**Table 3:** Localization scores for the 3 test conditions (BIN, LEO and REO) and the test–retests (S1–S3).

Condition	S1	S2	S3	Correlation
**BIN**	0.05 ± 0.22 [0; 1]	0.0 ± 0.0 [0; 0]	0.05 ± 0.22 [0; 1]	S1–S2: 0.17, ns S1–S3: 0.46, * S2–S3: 0.53, *
**LEO**	0.40 ± 0.60 [0; 2]	0.95 ± 0.76 [0; 3]	0.60 ± 0.50 [0; 1]	S1–S2: 0.07, ns S1–S3: 0.47, * S2–S3: 0.24, ns
**REO**	0.65 ± 0.93 [0; 3]	0.65 ± 0.59 [0; 2]	0.60 ± 0.82 [0; 2]	S1–S2: 0.11, ns S1–S3: 0.15, ns S2–S3: 0.39, ns

Localization score indicates the number of speakers between the source speaker and the designated speaker. Speakers were separated by 30 degrees. Correlation was assessed by Pearson’s r and p. ns: not significant, * *p* < 0.05.

## Data Availability

Data kept on a local secured database. Available on demand to the authors.
